# Cardiac autonomic dysfunction in chronic stroke women is attenuated after submaximal exercise test, as evaluated by linear and nonlinear analysis

**DOI:** 10.1186/s12872-015-0099-9

**Published:** 2015-09-29

**Authors:** Juliana Valente Francica, Aline Bigongiari, Luís Mochizuki, Kátia Bilhar Scapini, Oscar Albuquerque Moraes, Cristiano Mostarda, Erico Chagas Caperuto, Maria Cláudia Irigoyen, Katia De Angelis, Bruno Rodrigues

**Affiliations:** Human Movement Laboratory, São Judas Tadeu University (USJT), São Paulo/SP, Brazil; School of Arts, Sciences and Humanities, University of São Paulo, São Paulo/SP, Brazil; Hypertension Unit, Heart Institute (InCor), Medical School of University of Sao Paulo, São Paulo/SP, Brazil; Federal University of Maranhao (UFMA), São Luiz/MA, Brazil; Translational Physiology Laboratory, Universidade Nove de Julho (UNINOVE), São Paulo/SP, Brazil; Faculty of Physical Education, University of Campinas (UNICAMP), Av. Érico Veríssimo, 701. Cidade Universitária “Zeferino Vaz”. Barão Geraldo, Campinas, SP. CEP 13.083-851 Brazil

**Keywords:** Stroke, Autonomic nervous system, Spectral analysis, Symbolic analysis, Exercise test

## Abstract

**Background:**

We evaluated cardiac autonomic modulation in women with chronic ischemic stroke (at least 4 years post-stroke) at rest and in response to submaximal exercise test.

**Methods:**

Fourteen post-stroke women (S group) and 10 healthy women (C group) participated in this study. Autonomic modulation (using linear and nonlinear analysis), blood pressure and metabolic variables at rest were evaluated immediately after the exercise test and during the recovery period (20 min). All participants underwent submaximal exercise test on cycle ergometer with gas analysis.

**Results:**

At rest, the S group displayed higher lactate concentration, systolic (SBP) and diastolic blood pressure (DBP) values when compared to C group. Furthermore, the S group had lower heart rate variability (HRV) in time domain (SDNN: S = 30 ± 5 vs. 40 ± 8 ms; rMSSD: S = 14 ± 2 vs. C = 34 ± 3 ms), decreased high frequency band of pulse interval (S = 8.4 ± 2 vs. 33.1 ± 9 %) and 2V pattern of symbolic analysis (S = 17.3 ± 1 vs. 30 ± 3 %) (both indicators of cardiac vagal modulation) when compared to C group. Immediately after exercise, S group presented higher values of lactate, SBP, DBP and double product when compared to C group, as well as decreased heart rate recovery (HRR) measured at the first, second and third minutes. At recovery time, all HRV parameters in time and frequency domains improved in the S group; however, HF band remained lower when compared to C group.

**Conclusions:**

After the exercise test, women with chronic stroke presented reduced heart rate variability, reduced cardiac vagal modulation, as well as reduced HRR, while displayed an improvement of heart rate variability and cardiac vagal modulation when compared to their baseline. These results reinforce the importance of a physically active lifestyle for cardiovascular autonomic disorders observed in chronic stroke women.

## Background

Cardiovascular and cerebrovascular diseases are the major cause of mortality worldwide. In developed countries, stroke is the third leading cause of death. In USA, on average, every 40 s someone has a stroke, with women having a higher lifetime risk of stroke than men (each year ~55 000 more women than men have a stroke event) [1].

Stroke is also the leading cause of serious long-term disabilities [1], and further cardiac disease has been found to occur in up to 75 % of stroke survivors. In individuals with stroke, cardiac comorbidities may complicate the course of disease and contribute to early mortality [2]. Alterations initiated by cerebrovascular disease may negatively change the autonomic function and lead to cardiac impairment; or they may lead to a cerebral event, thus making more severe the existing autonomic dysfunction associated with cardiovascular risk factors [3, 4].

In the acute post stroke phase, individuals show autonomic imbalance characterized by decreased vagal modulation and increased sympathetic cardiac modulation [3, 5, 6]. This autonomic imbalance may contribute for end-organ damage, predispose to cardiovascular events [7] and it is correlated with the severity of neurological deficits and disability [8]. However, little is known about cardiac autonomic changes in patients after chronic stroke, particularly in women.

Heart rate variability analysis (HRV) is a well-reputed noninvasive method used to assess the autonomic modulation of the heart. This method allows the sympathetic and parasympathetic handles of the autonomic nervous system to the heart to be evaluated [9]. Exercise tests may also be an important tool to evaluate autonomic cardiovascular modulation and its responsiveness. In this sense, measuring heart rate recovery (HRR) after exercise tests may reveal the extent of reactivation of the vagal activity [10, 11]. Furthermore, studies have demonstrated that HRR is associated with short-term heart rate variability, and both have been associated with increased risk for cardiovascular events and sudden death [12–15].

Thus, given that stroke is more prevalent in women than in men, and that residual autonomic consequences of the stroke may influence prognosis, the aim of this study was to evaluate cardiac autonomic modulation in women with chronic stroke (at least 4 years of diagnosis) at rest and in response to submaximal exercise test through linear and nonlinear analyses.

## Methods

### Subjects

Fourteen women with at least 4 years after ischemic stroke diagnosis (S group) were recruited from the Neurological Physiotherapy Clinic of Sao Judas Tadeu University, along with 10 sex and age-matched control (C group) subjects from the surrounding area. Participants met the following eligibility criteria: (1) 50–70 years old; (2) sedentary, with no changes in physical activity over the previous 3 months; (3) non-obese; (4) non-alcoholic; (5) nonsmokers; (6) diagnosis of a first-time, ischemic front-parietal stroke which had occurred at least 4 years before enrollment; (7) able to walk (with or without an assistive device); (8) not using beta blockers (since this class of medications affects cardiovascular and autonomic response to exercise testing); (9) the possible presence of hypertension was not considered as a sufficient ground for excluding the patients from the study. Subjects were excluded if they had suffered from a recent cardiac event, acute cardiac or renal failure, or if they were regular smokers.

The study was conducted in accordance with Declaration of Helsinki. All subjects signed an informed consent form for this study, which was approved by the Ethical Research Committee of the Sao Judas Tadeu University (protocol number CEP-USJT: 383.800).

### Measurements

Subjects were instructed to avoid alcohol and caffeinated beverages for the preceding 24 h of evaluations, which were performed in the morning. Age and race were self-reported, and medications use, clinical history, as well as lifestyle habits were determined using standard questionnaires.

Bioelectrical impedance measure of body composition (Biodynamics® - 450 BIA) was carried out and body mass index was determined (BMI). The following experimental sequence was then adopted:

1. Baseline period: (i) blood pressure (BP) and heart rate (HR) measurements; (ii) RR interval registration; and (iii) metabolic variables evaluation (lactate and glucose).
2. Submaximal exercise test protocol.
3. Immediate post-exercise test: (i) assessment of BP and HR; (ii) metabolic variables evaluation.
4. Recovery period (20 min after the end of exercise test): (i) assessment BP and HR; (ii) recordings of RR interval for the 20 min of recovery; and (iii) assessment of metabolic variables

#### Blood pressure and metabolic measurements

At baseline, BP was measured by auscultation with the volunteers sitting and at rest. Three consecutive systolic (SBP) and diastolic BP (DBP) evaluations were carried out after 10-min rest period, with at least a 2-min interval between each one [16]. During submaximal exercise test and at the recovery period, BP was assessed once every 2 min. Double product (DP) was calculated by multiplying the HR and SBP.

The levels of lactate and glucose in the blood were measured at baseline, immediately after post-exercise test, and during recovery periods using a point of care hand-held lactate analyzer (Accutrend, Roche Diagnostics®, Mannheim, Germany). The analyzer is a small, battery-powered, reflectance photometer with a turnaround time of 60 s, which uses chemistry test strips on which a drop of blood is applied.

#### Autonomic evaluation by linear and nonlinear analyses

RR interval was continuously recorded for 20 min during both baseline and recovery, using a Polar® S-810i. The spectrum resulting from the Fast Fourier Transforms (FFT) modeling is derived from all the data present in the recorded signal; it includes the entire signal variance, regardless of whether its frequency components appear as specific spectral peaks or as non-peak broad band powers [17]. RR interval variability was evaluated in time and frequency domains. Spectral power for low (LF: 0.03–0.15 Hz) and high (HF: 0.15–0.4 Hz) frequency bands was calculated by means of power spectrum density integration within each frequency band width, using a customized routine (MATLAB 6.0) [18].

A symbolic analysis was carried out according to the approach previously described and validated by Porta [19]. However, the data were adjusted for 3 groups instead of 4, following Guzzetti et al. [20]. For this method, the same 5 min of iRR selected recording was used. Then, a coarse graining approach based on a uniform quantization procedure was used to transform the iRR series into a sequence of symbols. The length (L) was kept fixed in all analyses. The full range of the sequences was uniformly spread over 6 levels (from 0 to 5), and patterns of length L = 3 were constructed. Therefore, each subject and each experimental condition had its own range of iRR intervals. The Shannon entropy of the distribution of the patterns was calculated to provide a quantification of the complexity of the pattern distribution. All possible patterns (i.e., 216) were grouped without any loss into 3 families referred to as (1) patterns with no variation (0V; i.e., all 3 symbols were equal), (2) patterns with 1 variation (1V; i.e., 2 symbols were equal and the remaining symbol was different), and patterns with 2 variations (2V; i. e., all symbols were different from the previous ones) [20].

#### Submaximal exercise test protocol

The exercise test was performed on a cycle ergometer (Ergometric® 6.0). According to Tang et al. [21] and Billinger et al. [22], a cycle ergometer may be an alternative to a greater subset of the stroke survivor population because of the seated support and feet affixation in the pedals, which increase safety. A 3 min warm up was previously performed, at 10 Watts · min^−1^, without charge. This was followed by a ramp protocol of 10 Watts · min^−1^, with increments of 0.3 kp every minute, until it reached 80 % of maximal HR age predicted (using the formula 220 bpm - age), or by request of the subject. When any of these parameters was reached, the charge was removed and the protocol was finalized after 3 min cool-down at 10 watts. During exercise, HR was monitored by simultaneous 12-lead electrocardiogram (Wincardio, Micromed®) and BP, assessed each 2 min. The expired gases were continuously analyzed with metabolic analyzer VO2000 (NedGraphics®, USA). Peak of maximal oxygen consumption (VO_2_peak) was determined as the mean for an integral number of breaths over the final 20 s of the incremental phase.

Baseline HR was determined by the HR recorded as participants were standing and before the initiation of the treadmill test. Peak HR was determined as the highest value recorded across exercise protocol. HR recovery (HRR) were calculated as the difference between peak HR and HR at 1, 2 and 3 min after test cessation (HRR1’, HRR2’, HRR3’, respectively).

### Statistical analyses

Statistical analyses were performed with SPSS software (Version 20.0 for Windows; SPSS Inc., Chicago, USA). Data are presented as mean ± standard deviation (SD). Repeated-measures ANOVA was used to test changes at the baseline, post-exercise and recovery periods. One way ANOVA was applied to compare measurements between the groups at the same period. Post-hoc analysis was performed with Bonferroni test. Statistical significance was set at *P* < 0.05.

## Results

Subject profiles, medication use and associated comorbidities are shown in Table 1. We observed that 57 % of S group had previously been diagnosed with hypertension. There were no significant differences in age, body mass index, lean and fat body mass between the groups, while VO2 peak was smaller in S than in C group (Table 1).

**Table 1:** Participant characteristics

Parameters	C	S
Age (years)	61 ± 5	57 ± 6
Time after stroke (months)	-	60 ± 10
BMI (Kg/m^2^)	26.1 ± 3.3	29.3 ± 3.7
Lean Mass (%)	70.8 ± 6	65.2 ± 8
Fat Mass (%)	26.2 ± 5	34.1 ± 6
VO_2_ peak (mL/Kg^−1^/min^−1^)	37.8 ± 6	29.6 ± 4*
Medications (n)		
ACE inhibitor	3	7
HMG-CoA reductase inhibitor	2	9
Diuretic	4	1
Acetylsalicylic acid	-	10
Associated Comorbidities (n)		
Hypertension	3	8
Dyslipidemia	1	5

Values expressed as mean ± SD. C: control group; *S* stroke group, *BMI* body mass index, *VO*_*2*_ *peak* peak of oxygen consumption during submaximal exercise. *P < 0.05 vs. C

### Metabolic and hemodynamic responses to submaximal exercise test

During baseline evaluation, S group displayed higher lactate concentration, systolic and diastolic blood pressure values when compared to C group (Table 2). BG and DP at baseline were similar in both S and C groups. Immediately after exercise (post-exercise period), S group presented higher values of lactate, systolic and diastolic blood pressure, and DP when compared to C group and baseline evaluation.

**Table 2:** Metabolic and hemodynamic variables at baseline, immediately after exercise (post-exercise) and after 20 min of recovery

	Baseline		Post-exercise		Recovery	
	C	S	C	S	C	S
BG (mg/dl)	97 ± 5	99 ± 8	90 ± 9	90 ± 8	98 ± 7	91 ± 10
Lactate (mg/dl)	2.5 ± 0.6	4.0 ± 0.4*	3.5 ± 0.5	5.4 ± 0.5*†	2.7 ± 0.6	3.8 ± 0.8‡
SBP (mmHg)	109 ± 10	126 ± 9*	114 ± 8	138 ± 12*†	109 ± 6	125 ± 9*‡
DBP (mmHg)	76 ± 7	84 ± 5*	75 ± 8	89 ± 6*	76 ± 5	82 ± 8‡
DP (mmHg/bpm)	7980 ± 624	9393 ± 765	11871 ± 492†	13326 ± 952*†	8264 ± 802‡	10452 ± 919*‡

Values expressed as mean ± SD. *C* control group, *S* stroke group, *BG* blood glucose, *SBP* systolic blood pressure, *DBP* diastolic blood pressure, *DP* double product.*P < 0.05 vs. C at same time evaluation; †*P* < 0.05 vs. baseline in the same group; ‡*P* < 0.05 vs. post-exercise in the same group

During the recovery period, only systolic blood pressure and DP values remained higher in the S group when compared to the C group, while lactate and diastolic blood pressure were similar between the groups (Table 2). Furthermore, metabolic (except for BG) and hemodynamic parameters were reduced in the S group at the recovery period when compared to their post-exercise evaluation.

### Heart rate and heart rate recovery

**Fig. 1:**
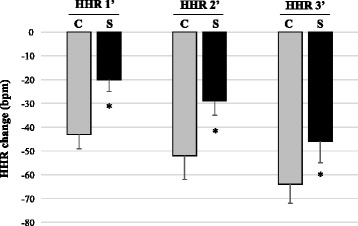
Heart rate recovery changes at the 1st, 2nd, and 3rd minutes in the Control (C) and Stroke (S) groups. *P < 0.05 vs. C at same time evaluation

No differences were observed in HR at baseline, peak of exercise test, and recovery periods between the groups (Table 3). However, HR values in the 1^st^ and 2^nd^ minutes after exercise test cessation remained higher in the S group when compared to C group, while HR values remained unchanged in the 3^rd^ minute after the end of test in all groups. In addition, HRR values in the 1^st^ (20 ± 5 vs. 43 ± 6 bpm; *P* < 0.001), 2^nd^ (29 ± 6 vs. 52 ± 10 bpm; *P* = 0.001) and 3^rd^ minutes (46 ± 9 vs. 64 ± 8 bpm; *P* = 0.105) were reduced in S group when compared to C group (Fig. 1).

**Table 3:** Heart rate responses (bpm) to submaximal exercise test

Parameters	C	S	*P* values
Baseline	73 ± 10	75 ± 15	0.837
Peak HR	153 ± 12†	144 ± 15†	0.688
HR 1′	110 ± 15†	124 ± 18†*	0.028
HR 2′	101 ± 15†	115 ± 12†*	0.001
HR 3′	89 ± 17	98 ± 13	0.093
Recovery	76 ± 18	82 ± 10	0.083

Values expressed as mean ± SD. *C* control group, *S* stroke group, *HR* heart rate, *HRR* heart rate recovery. *P < 0.05 vs. C at same time evaluation; †*P* < 0.05 vs. baseline in the same group

### Cardiac autonomic modulation

Regarding heart rate variability in time domain, the S group had lower values of VarRR, SDNN, rMSSD, and pNN50 than C groups at the baseline period. Throughout the recovery period, the S group had higher VarRR, SDNN, pNN50 and rMMSD when compared to their baseline evaluation (Table 4). Furthermore, at recovery time, the S group presented similar SDNN and rMSSD values when compared to C group.

**Table 4:** Heart rate variability in the time and frequency domain at baseline and during recovery from exercise protocol

	Baseline		Recovery	
Parameters	C	S	C	S
Time Domain				
VarRR (ms^2^)	1152 ± 39	717 ± 44*	1346 ± 130	890 ± 66*†
SDNN (ms)	40 ± 8	30 ± 5*	41 ± 6	44 ± 8†
rMSSD (ms)	34 ± 3	14 ± 2*	42 ± 4	35 ± 3†
pNN50 (%)	8.7 ± 1.0	2.2 ± 0.5*	17.3 ± 4.2†	7.5 ± 1.6*†
Frequency Domain				
LF (ms^2^)	266 ± 80	216 ± 35	317 ± 81	244 ± 33†
LF (%)	23 ± 6	12 ± 4	21 ± 5	25 ± 8†
HF (ms^2^)	384 ± 75	55 ± 13*	533 ± 80	154 ± 30*†
HF (%)	33.1 ± 9	8.4 ± 2*	38.2 ± 6	16.3 ± 3*†
Symbolic analysis				
0V (%)	21 ± 3	39.9 ± 2	24 ± 3	30.0 ± 3†
1V (%)	49 ± 1	42.5 ± 1	48 ± 2	45 ± 2
2V (%)	30 ± 3	17.3 ± 1	28 ± 4	25 ± 3†

Values expressed as mean ± SD. *C* control group, *S* stroke group, *VarRR* variance of RR interval, *SDNN* standard deviation of the RR interval, *rMSSD* root-mean square of differences of successive RR intervals, *pNN50*% of differences of adjacent RR intervals > 50 ms, *LF* low frequency band in absolute and normalized values, *HF* high frequency band in absolute and normalized values, *LF/HF* low frequency/high frequency ratio; patterns with no variation (0 V; i. e., all 3 symbols were equal), (2) patterns with 1 variation (1 V; i. e., 2 symbols were equal and the remaining symbol was different), and patterns with 2 variations (2 V; i. e., all symbols were different from the previous ones) *P < 0.05 vs. C; †*P* < 0.05 vs. baseline in the same group; ‡*P* < 0.05 vs. post-exercise in the same group

**Fig. 2:**
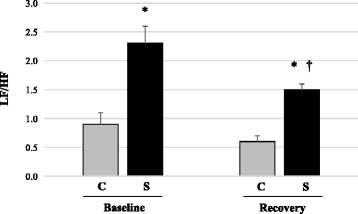
Autonomic balance (LF/HF ratio) in the Control (C) and Stroke (S) groups. *P < 0.05 vs. C at same time evaluation; †*P* < 0.05 vs. baseline in the same group

In frequency domain (Table 4) at baseline evaluation, the S group had a decrease in HF band (absolute and normalized values) when compared to the C group. Throughout the recovery period, the S group displayed increased LF band (absolute and normalized values) and HF band (absolute and normalized values) when compared to the baseline evaluation. However, despite the increase in HF band in the S group, these values remained lower than those found for the C group. Similarly, LF/HF ratio was increased in S group when compared to C, both at baseline (2.3 ± 0.3 vs. 0.9 ± 0.2), and during recovery (1.5 ± 0.1 vs. 0.6 ± 0.1). However, it should be noted that the LF/HF ratio was reduced in S group when compared to the baseline evaluation (Fig. 2).

The results of symbolic analysis are shown in Table 4. At baseline evaluation, the 0V pattern, which indicates sympathetic modulation, was increased, while 2V pattern, an indicator of parasympathetic modulation, was reduced in S group when compared to C group. During recovery, both parameters, 0V and 2V, were increased in S group when compared to their initial evaluation, reaching similar values to C group.

## Discussion

The main finding of our study is that patients with chronic stroke (5 years in average) presented decreased heart rate variability, autonomic imbalance and impaired cardiac vagal modulation, as measured by linear and nonlinear analysis. Other important findings in women with chronic ischemic stroke, when compared to controls, include: (1) reduced values of VO_2_ peak and higher levels of blood lactate; (2) decreased HRR measured at 1, 2 and 3-min after submaximal exercise test; (3), improved hemodynamic and cardiac autonomic parameters during the recovery period (20 min after the end of exercise test).

Most of the survivors had residual disabilities caused by stroke, such as hemiparesis and spasticity, while total recovery was much less frequent. Activity limitations were demonstrated by the reduced ability to perform daily tasks and basic self-care, leading the individual to chronic sedentary behavior [22]. Regarding this issue, in a study conducted by Gadidi et al. [23], the percentage of subjects reporting some activity limitation 4 years post stroke was 42.3 %, while 28.2 % pointed to less severe limitations and 78.1 % felt they had not fully recovered. Although we did not classify individuals according to their self-reporting of activity limitations, we observed that VO_2_ peak was reduced by 21 % in stroke women, and blood lactate levels were higher at rest and after exercise test when compared to the controls, which seems to indicate physical deconditioning and sedentary lifestyle on stroke survivals.

Physical deconditioning usually leads to physiological and metabolic changes in the paretic muscle. These changes are characterized by decreased blood flow, increased lactate production, increased muscle glycogen utilization and decreased ability of fatty acid oxidation. In addition, changes in muscle fibers during exercise were observed: active paretic muscle activated glycolytic type II fibers to initiate contraction, while the non-paretic muscle recruited primarily type I fibers. These changes usually lead to disuse and causes decreased oxidative metabolism, low resistance to aerobic exercise, early fatigue, sedentary lifestyle and deconditioning [24–26].

Blood pressure values were higher in S group (at rest, in the post exercise and recovery period) when compared to C group. Although 57 % of S group individuals had prior history of hypertension, blood pressure values observed in these patients were within normal parameters, showing that the pressure levels were controlled [16]. Similarly, Dütsch et al. [27] have found no alterations in blood pressure in stroke patients 30 months after stroke. In addition, since that double product seems to be an indirect predictor of myocardial oxygen consumption [28, 29], our findings suggest that women with chronic stroke require higher myocardial work when compared to the control group.

In the present study, even after nearly five years post-stroke, women presented reduced time domain parameters (VarNN, SDNN, rMSSD and pNN50) of heart rate variability at rest. Similarly, Muslumanoglu et al. [5] have observed reduced values for VarRR, SDNN and pNN50 in the post-stroke acute phase. Dütsch et al. [27] have shown that post–acute stroke patients presented parasympathetic cardiac deficit and higher LF/HF than age-and sex-matched controls. In our study, women with stroke showed a decrease in both HF band of spectral analysis and in 2V pattern of symbolic analysis. These two measurements serve as indicators of both vagal cardiac modulation and autonomic imbalance, as demonstrated by the LF/HF ratio.

Since the 80s, researchers have indicated that physical inactivity is associated with negative changes in autonomic nervous system. Reduction in blood volume affects cardiac stroke volume; as such, maintaining oxygen delivery [30] requires an increase in heart rate triggered by increased sympathetic and reduced parasympathetic activity to the sinoatrial node Furthermore, studies with experimental models of physical inactivity, associated with a sedentary lifestyle or extreme forms of inactivity with bed rest or spaceflight, have pointed to a decrease in parasympathetic drive and an increase in the sympathetic tonus to heart [See for review, 31]. These findings may explain, at least in part, the autonomic imbalance observed in chronic stroke individuals observed in the present study.

Some stroke subjects (57 %) were diagnosed with hypertension before the event, and most of them were being treated with angiotensin-converting enzyme (ACE) inhibitors. Most antihypertensive drugs induce a rearrangement of the autonomous nervous system. It is well-established that central sympatholytic agents and beta-blockers induce amplified inhibitory effects on sympathetic activity. Furthermore, angiotensin-converting enzyme inhibitors and angiotensin II receptor antagonists may also promote reduction of sympathetic tone, although to a lesser extent. On the other hand, other compounds may either be neutral or may play unfavorable role on the sympathetic nervous system, such as long-acting calcium channel blockers, diuretics, and short-acting calcium channel blockers, respectively [32–34]. Although it is not possible to differentiate whether autonomic dysfunction, as displayed by the stroke group, was a result of ischemic cerebral event, previous hypertension, or both, we suggest that the residual motor disability triggered by stroke may be the main reason, since blood pressure levels were controlled and within normal limits.

Regarding predictive values of heart rate variability, it is well established that alterations in these parameters may predispose individuals to arrhythmias and cardiac events [35–37], being associated with several diseases, e.g., myocardial infarction [38, 39] heart failure [40], diabetes [41] and stroke [42, 43]. Moreover, heart rate variability has been found to be a predictor of stroke in subjects aged 55–70 years and without cardiovascular disease [44].

In addition to autonomic modulation analysis, we observed that women with chronic stroke showed a decrease in HRR measured at 1, 2 and 3 min after the end of test when compared to controls. These results are indeed significant, since the decreased HRR at 1, 2 and 3 min after exercise is mainly a result of impaired vagal reactivation, a predictor of cardiovascular events [45–47]. Furthermore, decreased vagal tone is found in several conditions and it is generally associated with poor cardiovascular prognosis [48–50]. In fact, HRR after exercise seems to be correlated with heart rate variability in the early recovery phase after submaximal exercise [51, 52], reinforcing our findings.

To the extent that the parasympathetic modulation is deteriorated, achieving an adequate heart rate during exercise has proved challenging, as well as returning to baseline values. Thus, the impairment of vagal function is detectable not only for heart chronotropic incompetence (an aspect which has not been covered in this study), but also for the recovery of heart rate immediately after exercise.

According to Mravec [53], afferent and efferent vagal pathways may affect several mechanisms involved in the onset and progression of stroke. One of the mechanisms is the central and peripheral inflammation, which may either lead to stroke or be stroke-induced. Reduced vagal activity mediated by a decrease in cholinergic anti-inflammatory pathway may be accompanied by an increase in the pro-inflammatory status and may represent a risk factor for stroke. Yet, stroke may alter vagal immunoregulatory functions and lead to inflammatory reactions in the peripheral tissues and brain [53].

Since the presence of residual motor disabilities may lead to a chronic condition of physical inactivity, and consequently to an exacerbated autonomic dysfunction, chronic stroke patients remain at high risk for cardiovascular events, including another stroke, and this should not be overlooked. In this sense, several experimental and clinical studies have demonstrated that exercise can improve cardiovascular autonomic function in stroke subjects within a short time after the event [54–58]. In our study, we demonstrated that, although some parameters of heart rate variability, i.e., VarNN, pNN50 and HF, remained lower than those found for controls subjects, improvement was actually detected in all time and frequency domain parameters, as well as in the symbolic analysis in the stroke group at recovery after submaximal exercise test. These data suggest that aerobic exercises, if well conducted and carefully monitored, may be an effective non-pharmacological strategy to improve heart rate variability and cardiac vagal modulation in stroke women, even after a chronic period after the event.

This study has limitations that deserve comments. First, although we did not divide patients according to injury side, they had similar patterns of reduced parasympathetic modulation, both at baseline and in the recovery period. Second, the lack of a hypertensive control group when more than half of patients with stroke had a history of hypertension does not allow us to distinguish the participation of each of these diseases on autonomic dysfunction presented by the stroke group. However, this issue was not the focus of this study. Another limitation lies in the fact that our study population was undersized, i.e., it was not large enough (14 chronic stroke women and 10 controls) to enable us to draw conclusions suitable for extending and generalizing our inferences and results. In fact, the minimum sample size to promote a power of 80 % in paired analyses was in C group *n* = 12 and in S group *n* = 16. This calculation has been carefully considered by the G * Power 3.2.1 software. Finally, although we excluded women who were taking beta-blockers, the presence of comorbidities (as hypertension) and the medications use may had some influence on cardiovascular autonomic modulation of the evaluated individuals.

## Conclusion

In conclusion, we found that women with chronic stroke (5 year post-stroke, in average) presented negative changes in lactate, aerobic capacity and autonomic modulation, more specifically decreases in vagal component of heart rate variability and symbolic analysis. Furthermore, in response to submaximal exercise test, women with stroke showed impaired heart rate recovery immediately after the exercise test, probably due to reduced vagal modulation. These results highlight the importance of detecting and preventing the loss of parasympathetic function in patients after a chronic stroke, since such impairment may be an important risk factor for new cerebrovascular or cardiovascular events. On the other hand, in the recovery period after submaximal exercise test, autonomic modulation was improved when compared to baseline levels of stroke women, emphasizing the importance of avoiding the physical deconditioning in women after stroke. It is important to emphasize that further studies with larger populations are needed in order to dissect out, in subgroups analyses, the contribution of the different co-factors to the observed heart rate variability alterations.

## Abbreviations

ACE: Angiotensin converting enzyme
BG: Blood glucose
BMI: Body mass index
BP: Blood pressure
C: Control group
DBP: Diastolic blood pressure
DP: Double product
FFT: Fast fourrier transformer
HF: High frequency band
HR: Heart rate
HRR: Heart rate recovery
HRV: Heart rate variability
LF: Low frequency band
pNN50: Percent of differences of adjacent RR intervals > 50 ms
rMSSD: Root-mean square of differences of successive RR intervals
S: Stroke group
SBP: Systolic blood pressure
SDNN: Standard deviation of the RR interval
VarRR: Variance of RR interval
VO2 peak: Peak of oxygen consumption
